# The Evolution of Aesthetic Medicine: Exploring the Intersection of Social Psychology, Technology, and Aesthetic Medicine

**DOI:** 10.1093/asjof/ojaf015

**Published:** 2025-03-13

**Authors:** Steven Dayan, Berno Bucker, Jacques van der Meulen, John St John Blythe, Patricia Ogilvie, Damon Caiazza, Maria Musumeci, Michael Silberberg

## Abstract

A wealth of information can be garnered from the face, such as current emotional state, perceived overall health, personality traits, and even attributed capabilities. It is well understood within aesthetic medicine that minimally invasive treatments positively influence how a patient feels about themselves from an emotional and physical standpoint; however, a growing body of literature suggests that facial aesthetic treatments can also impact how an individual is perceived by others. First impressions, which are formed within milliseconds of viewing a new face, allow us to intuit information about the person and anticipate their intentions. Multiple theories aim to explain why and how these initial social impressions are formed from relatively simple visual cues, and increasing evidence suggests that augmentation of facial appearance influences how others form first impressions. In this article, we discuss theories underlying the importance of first impressions and how minimally invasive aesthetic treatments can affect first impressions and social attributions. We also discuss how advancements in technology can enhance consultations and how technology may expand the world of aesthetic medicine to patients who had not previously considered engaging with aesthetic treatments.

**Level of Evidence: 5 (Diagnostic):**

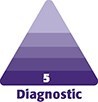

The face can convey a wealth of information about an individual, from simple categorical assessments to perceived overall health, personality traits, and attributed skills. For fast and economic assessment, humans have developed an elaborate sensory system that rates facial characteristics as a proxy for broader impressions of traits and behaviors. Multiple studies have demonstrated that facial aesthetic treatments positively influence the physical and emotional well-being of patients.^1-4^ In controlled studies, the effectiveness of aesthetic treatments is often determined through clinical assessments of the degree of anatomical change (eg, physical change from baseline in severity of upper facial lines) and patient-reported outcomes, including quality of life data. Similarly, in reconstructive patients (eg, those who experienced a traumatic injury necessitating facial reconstruction), adjunctive treatment with minimally invasive injectables can improve the patient's satisfaction with their appearance, improve their quality of life, and reduce psychosocial distress.^5^ Often, the emphasis is placed on how an aesthetic treatment makes the individual feel about *themselves*.

But aesthetic medicine can offer advantages that go beyond physical improvement, and minimally invasive aesthetic treatments can have a positive *social* impact on patients.^1,3,6-8^ Indeed, one's outward appearance factors heavily into how they are perceived by others and how they might interact with others. When viewing the face of another individual, people form a first impression and almost instantaneously ascribe social attributions (ie, personality traits, potential behavioral outcomes) based on the appearance of that individual's face, whether their face is expressing an emotion or at rest. By incorporating a discussion of socially based treatment outcomes into the consultation, aesthetic practitioners can better help patients attain their desired outcome. Furthermore, the demonstrated ability of injectables to positively impact third-party impressions may lead individuals who have never previously considered treatment to be open to aesthetic intervention.

In this article, we discuss the psychology of first impressions, evidence for improved first impressions following aesthetic treatment, and how technology can be incorporated into consultations to educate prospective patients on socially based treatment outcomes.

## PSYCHOLOGY OF FIRST IMPRESSIONS

First impressions are formed rapidly; 1 study reported that only <100 ms were required for personality traits such as extraversion to be inferred from an initial impression.^9^ Evaluation of others based on their facial appearance is inevitable, and the brain makes split-second judgments on personality traits when seeing a new face. Multiple theories exist to attempt explaining why and how initial social impressions are generated, including the overgeneralization theory and valence-dominance model.

The overgeneralization theory states that the evaluation of facial appearance by our emotional detection system is often overgeneralized to cues that guide future behavior. For example, cues about the emotional state of a person (eg, a smile) are overgeneralized, so people whose natural facial appearance resembles a smile leads to these people being judged as having a happier or friendlier personality. Also, encountering a person while smiling can be overgeneralized to assuming this person is always smiling. Similar overgeneralizations can be made when someone has a facial appearance that resembles a particular emotion (the emotion face overgeneralization), babies (the baby face overgeneralization), or a particular known identity (the familiar face overgeneralization).^10^ These singular events and social judgments are then viewed as an invariable rule of personality that dictates future interactions,^10-16^ although the effect sizes of these overgeneralizations can be small.^9^

An alternative theory, Oosterhof and Todorov's valence-dominance model, posits that social judgments can be mapped to 2 underlying dimensions: valence (ie, trustworthiness) and dominance.^17^ From an evolutionary standpoint, it is important to anticipate others’ intentions toward us (ie, trustworthiness) and assess the others’ capability to pursue their intentions (ie, dominance) to increase survival and reproduction rate. Importantly, this model has been shown to generalize across multiple cultures.^18-20^

In practice, facial judgment influences a variety of perceptions and outcomes. For example, physical appearance (eg, rounder vs more square face) contributes to perceptions of attractiveness, age, and gender.^10,21-23^ There are also universal inferences (ie, evolutionary mechanisms of perceived reproductive fitness), cultural specificity (face typicality, or how similar 1 face is to an average face in a cultural or ethnic group), and idiosyncratic factors (eg, resemblance to self or significant others) that affect how initial first impressions may be formed and then acted upon.^23,24^ First impressions also influence behavior. Without conscious awareness, interactions with others are influenced by first impressions and, in line with the self-fulfilling prophecy, people might *believe* in the trait judgments formed from first impressions and align their behavior accordingly. Thus, it can be argued that our facial appearance can strongly influence the outcomes of social events, such as judgments of attractiveness influencing romantic relationships, judgments of competence influencing professional and financial success, and several other judgments based on facial attributes influencing predictions of social outcomes (eg, voting, investment, sentencing decisions, punishment severity).^24-31^

## THE INFLUENCE OF AESTHETIC MEDICINE ON SOCIAL PERCEPTIONS

A growing body of literature demonstrates that changing an individual's facial appearance (ie, with aesthetic treatments) can influence first impressions and social perceptions. An early study had nonphysicians (patients and general population) grade randomized preoperative and postoperative photographs of 14 patients who had received various facial surgeries (eg, rhinoplasty, blepharoplasty, chin implant) on multiple categories of first impressions. All categories were rated as higher for postoperative photographs, with the highest increases seen for attractiveness, attributed social skills, and attributed dating success.^1^ Similarly, multimodal treatment with neurotoxin, soft-tissue fillers, and eyelash growth treatment was associated with patients being rated as more attractive, successful, friendly, approachable, younger, and more socially adept.^3^ A recent cross-sectional online survey study investigated whether minimally invasive procedures in female patients affect 3 domains of facial information according to independent observers who do not have a background in aesthetic medicine: attractiveness (young, attractive, and healthy), competence (knowledgeable, competent, intelligent, confident, masculine, and dominant), and trustworthiness (friendly, approachable, aggressive, credible, honest, and trustworthy). There was significantly higher agreement that patients appeared competent, trustworthy, and attractive after treatment than before treatment. An exploratory domain of naturalness was not changed by treatment. All but 1 subdomain was significantly changed posttreatment; perceptions of dominance did not significantly change posttreatment, which authors proposed as being due to increased femininity (ie, significantly decreased “masculinity”).^6^ Although all patients in the study were female with low Fitzpatrick skin phototypes, and therefore it is not clear whether these findings are applicable to men, or women with higher Fitzpatrick skin phototypes, the results suggest that overall impressions are formed holistically, not necessarily from a single facial area, and that facial features are interconnected. Other studies have shown that treatment with hyaluronic acid injectables is associated with better overall first impressions, attractiveness, youthfulness, confidence, and happiness, as well as attributed social skills, athletic skills, and dating success.^7,8^ The importance of the quality of the treatment result also appears to influence first impressions, with higher ratings of perceived attractiveness for patients whose treatment results appeared more “natural-looking.”^7^ A limitation of these studies is that they often rely on static images, which may not capture relevant dynamic facial movement and may not directly translate to first impressions in a real social context.

Facial differences, either congenital or acquired, also impact how an individual is perceived by others and correction may positively influence first impressions. Asymmetries produced by facial paralysis are associated with more negative perceptions of affect and lower ratings of attractiveness,^32,33^ which eye-tracking studies suggest may be because of attentional bias toward the asymmetry of the paralyzed face (ie, the mouth at rest or smiling).^34^ Patients with dentofacial deformities are perceived as possessing lower degrees of positive personality traits, such as confidence and intelligence, and higher degrees of negative traits such as aggressiveness and brutality.^35^ Aesthetic medicine may help these individuals augment their appearance in a way that can potentially reduce stigmatization and negative interactions with others. Indeed, multiple studies have demonstrated that surgical treatment of facial differences is associated with more positive personality and trait assessments by others.^36-39^

Although most published literature describes how social perceptions are influenced by the correction of facial differences using *surgical* intervention, minimally invasive treatments may also be an effective tool. Indeed, a growing number of articles report and highlight improved levels of satisfaction in relation to facial appearance and quality of life compared with levels of satisfaction with their facial differences before adjunctive facial aesthetic treatments.^40-46^ This is a powerful finding because many patients with acquired facial differences have deep levels of facial dissatisfaction, anxiety, and depression. Partial or total resolution of their facial-related anxieties may not only be related to an improvement in facial appearance but the “corrective” results from injectables may erode the strong physical cues that stimulate the daily traumatic recollections and posttraumatic stress. However, published data on the effects of minimally invasive treatments in patients with facial differences are limited, and it is not currently known whether minimally invasive treatments can stand alone in meaningfully improving first impressions or social interactions, or whether these treatments need to be adjunctive to surgical methods.^47^ Additional research is warranted to better understand the role and relative effectiveness of minimally invasive vs surgical treatments in influencing first impressions in this population.

## THE GROWING ROLE OF MOBILE TECHNOLOGY AND ARTIFICIAL INTELLIGENCE IN AESTHETIC MEDICINE

As technology continues to advance, practitioners will have tools at their disposal to analyze how patients’ facial appearance may influence social perceptions as well as how specific aesthetic treatments might augment these social perceptions. Articles describing recent preliminary studies have reported on the use of artificial intelligence (AI), augmented reality, and/or machine learning for surgical planning of reconstructive patients,^48^ to predict facial morphology following orthognathic surgery,^49^ and to classify severity of facial wrinkles.^50^ AI-based applications have been used to show improvements in perceived attractiveness and age following aesthetic surgery procedures.^51-53^ This technology has also been used to show that bilateral brow lift surgery or facial rejuvenation surgery (facelift with or without brow lift, blepharoplasty, or fat grafting) decreases ratings of perceived sadness or anger with concurrent increases in detection of happiness.^54,55^

Published data on technology for assessing social outcomes of minimally invasive aesthetic treatments is lacking, and although the use of this technology is currently limited, it is growing in use. As we look to the future, the use of smartphone-based imaging coupled with AI creates a journey where a patient can capture a clinical-grade selfie using their smartphone, have an initial AI-powered assessment of facial features, select a treatment plan comprising minimally invasive treatments, visualize the before and after images of potential treatments, and assess outcomes across a wide array of clinical outcome assessment dimensions (eg, social perception). Providers are just beginning to leverage the power and potential of clinically validated technology in aesthetic medicine, and technological developments in the future will enable richer engagements between patients and technology and providers and technology, thus enriching the overall provider and patient experience.

## FROM THEORY TO CLINICAL PRACTICE

Patients may engage with aesthetic medicine to receive a specific treatment, to address a concern with an anatomical facial feature, or to address a concern with a social feature (eg, looking sad).^56^ Although healthcare professionals are trained to provide anatomical or technical solutions to aesthetic requests, recognizing and even addressing the social benefits that these solutions provide has only recently come to our attention. By becoming aware of the social aspects of what we radiate and the potential impact of our treatments on our patients’ social lives, aesthetic providers can better understand and cater to the needs and expectations of our patients. This also accounts for patients seeking reconstructive treatments (eg, lightening the appearance of scars, correcting injury-induced volume and/or shape deficits), where the social aspects of the face are traditionally considered secondary to physical optimization.

The social relevance of the above makes it important to discuss perceptions as part of a consultation. Awareness among aesthetic providers can be accomplished by sharing information through published articles, photographs, etc, about the importance of first impressions. Additionally, it is important that there is alignment between the outcomes desired by the patient and the goals of the provider (eg, natural-looking outcomes, improving how the patient feels about themself, noticeable differences in appearance). Recent technological developments (eg, treatment simulation) can help both the aesthetic provider as well as the patient in better decision-making regarding the applied treatments, resulting in higher patient satisfaction levels. AI-based solutions could also allow patients to “see” how minimally invasive aesthetic treatments may affect different social perceptions (eg, aesthetics, social, professional), or these AI-based solutions may allow physicians to tailor treatments to the desired outcome.

## CONCLUSIONS

Aesthetic medicine can have far-reaching benefits for patients that extend beyond beautification and rejuvenation, as aesthetic treatment can significantly influence third-party impressions and impact how treated individuals interact with others, thus leading to a socially transformative outcome. Furthermore, raised awareness of the benefits of minimally invasive aesthetic treatments beyond the physical improvement may open up these treatments to those who may not have previously considered them.
